# 电磁导航支气管镜联合径向探头支气管内超声在肺周围型病灶诊断中的应用

**DOI:** 10.3779/j.issn.1009-3419.2017.12.12

**Published:** 2017-12-20

**Authors:** 海涛 黄, 少慕 陈, 良斌 潘, 科 陈, 飞荣 姚, 海涛 马

**Affiliations:** 215006 苏州，苏州大学附属第一医院 Department of Thoracic Surgery, The First Affiliated Hospital of Soochow University, Suzhou 215006, China

**Keywords:** 电磁导航支气管镜, 径向探头支气管内超声, 肺周围型病灶, Electromagnetic navigation bronchoscopy, Radial probe endobronchial ultrasound, Peripheral pulmonary lesion

## Abstract

**背景与目的:**

随着高分辨计算机断层扫描（computed tomography, CT）的广泛应用和健康体检的普遍开展，大量周围型肺部病灶被发现，对临床诊断治疗提出了新的挑战。电磁导航支气管镜（electromagnetic navigation bronchoscope, ENB）和径向探头支气管内超声（radial probe bronchoscopy ultrasound, R-EBUS）是用于周围型肺部病灶诊断的新兴技术，本研究旨在探讨ENB联合R-EBUS对肺周围型病灶诊断中的应用价值。

**方法:**

2016年9月-2017年11月苏州大学附属第一医院胸外科应用ENB技术对18例患者的30处肺部周围型病灶进行了检查，术前制定导航计划，术中导航成功到达预定位置后使用R-EBUS确认病灶，依次使用穿刺针、细胞刷、活检钳进行病灶组织活检。

**结果:**

30处肺部周围型病灶，导航成功率为100%（30/30），阳性诊断率为90%（27/30）。手术时间为（95.61±28.74）min，每处病灶导航时间为（25.90±11.29）min，发生气胸1例，未见其他严重并发症。

**结论:**

利用ENB联合R-EBUS技术诊断肺周围型病灶具有较高的导航成功率和诊断阳性率，安全有效，值得进行临床推广。

## Abstract (en)

**Background and objective:**

With the application of high resolution computed tomography (CT), a large number of peripheral lung lesions were found. It put forward new challenge on clinical diagnosis and treatment for these peripheral lung lesions. Electromagnetic navigation bronchoscopy (ENB) and radial endobronchial ultrasound probe (R-EBUS) are new technologies used for the diagnosis of peripheral lung lesions. The aim of this study is to explore the application value of ENB combined with R-EBUS in the diagnosis of peripheral pulmonary lesions.

**Methods:**

From September 2016 to November 2017, eighteen patients with thirty peripheral pulmonary lesions in the First Affiliated Hospital of Soochow University were enrolled. The ENB was performed on these patients who were detected peripheral lung lesions by chest HR-CT. After successful navigation, the lesion's location was confirmed by R-EBUS, and specimens were acquired by needle aspiration, endoscopic cell brush and biopsy forceps.

**Results:**

A total of eighteen patients with thirty lesions were enrolled in this study, the navigation success rate was 100%, the positive rate was 90%. The mean operation time was (95.61±28.74) min, and navigation time for each lesion was (25.90±11.29) min, and pneumothorax was observed in 1 case.

**Conclusion:**

ENB combined with R-EBUS for the diagnosis of peripheral pulmonary lesions is safe and effective. This technique is worth promoting.

近年来，随着高分辨计算机断层扫描（computed tomography, CT）的广泛应用和健康体检的普遍开展，大量的肺部病变被发现，其中大部分为周围型病灶，对临床诊断治疗提出了新的挑战。以往，经皮肺穿刺是肺周围型病变最常用的方法，诊断准确率可达到90.00%^[1]^，但气胸发生率也高达25.00%^[2]^；而且经皮肺穿刺技术对于靠近中央气道及肺门大血管的病灶穿刺风险较大，临床应用局限性较大。电磁导航支气管镜（electromagnetic navigation bronchoscope, ENB）结合了现代电磁导航、虚拟支气管镜和三维CT成像技术，可以深入到达12级-14级支气管，实现肺部病灶的实时导航和精确定位。对于肺部周围型病灶的诊断，电磁导航支气管镜与经皮肺穿刺的诊断率接近，但气胸等并发症的发生率更低^[2]^。

苏州大学附属第一医院胸外科于2016年首次在江苏省将ENB技术应用于临床，并联合径向探头支气管内超声（radial probe bronchoscopy ultrasound, R-EBUS）诊断肺周围型病变。本研究通过对30例肺外周型病灶的导航操作总结了ENB联合R-EBUS技术的操作的经验体会，希望为该技术的推广和拓展应用提供参考。

## 1 资料与方法

### 1.1 研究对象

选取2016年9月-2017年11月苏州大学附属第一医院胸外科行ENB检查术的患者18例，合计外周性肺病灶30处。其中男性12例，女性6例，年龄52岁-82岁，中位年龄68岁。病灶位置分布如下：右上肺尖段1例，右上肺后段6例，右上肺前段5例；右下肺前基底段2例，右下肺内基底段1例，右下肺后基底段1例，右下肺外基底段1例；左上肺前段4例，左上肺后段1例；左下肺背段1例，左下肺后基底段3例，左下肺外基底段4例。所有入组患者均通过高分辨率胸部CT确认存在肺周围型病变，在完善术前检查后，确认无相关禁忌症后进行ENB检查。纳入标准：年龄≥18岁；心肺功能可以耐受检查；胸部CT明确诊断肺部结节且需行肺组织活检；预计经普通支气管镜无法到达的病灶；无明显麻醉禁忌症。排除标准：年龄＜18岁；心肺功能不全无法耐受检查者；凝血功能障碍者；不能耐受麻醉者。该研究通过苏州大学附属第一医院伦理委员会批准，且所有入组患者得到告知，并签署知情同意书。

### 1.2 研究方法

#### 1.2.1 ENB

电磁导航系统（美国Super Dimension公司）由5个部分组成：①一台安装inReach专用导航软件的电脑，用于在术前导入高分辨CT数据进行导航路径规划并导出到U盘。②一块可以发射电磁信号的电磁定位板，术中置于患者背部的手术床上，与放置在患者胸部的三枚磁极一起构成电磁场，覆盖患者胸部区域，提供电磁信号进行实时定位导航。③一根外径1.9 mm的定位导向管，导管前端连接有电磁传感器，能接受电磁信号，并可以通过控制手柄向360°的8个方向弯曲，深入到达肺内病灶。④一根内径2.0 mm的扩展工作通道，进行导航操作时定位导管插入扩展工作通道中，与其通过旋钮连接并一起进入纤维支气管镜的钳道，导航到达病灶后可解除连接并退出定位导向管，将扩展工作通道置于病灶部位，然后通过扩展工作通道置入R-EBUS检查病灶，并使用各种细胞刷、穿刺针和活检钳等工具对病灶进行组织活检。⑤一台电磁导航主机，连接电磁定位板、定位导管、电子支气管镜主机等设备，通过U盘导入术前制定的导航路径规划，将患者的虚拟支气管树以及导航路径叠加显示在触摸屏上，指导术者实施注册以及导航操作。

#### 1.2.2 R-EBUS

采用日本奥林巴斯公司产品，R-EBUS型号：UM-S20-17S，支气管镜主机型号：CV-190，支气管镜型号：1T180，通过支气管镜钳道携带导航定位导向管和扩展工作通道进入支气管。

### 1.3 术前准备

手术开始前将电磁定位板平放于手术床上，患者取去枕仰卧位，胸部置于电磁定位板上方的区域；胸骨角、双侧腋中线第八肋间放置3个磁极，形成等腰三角形，可根据患者体型适当调整磁极的位置，确保患者胸部和三个磁极完全置于电磁场中。静脉诱导全身麻醉，喉罩或气管插管通气。

### 1.4 具体操作步骤

①CT扫描：患者进行高分辨率胸部CT扫描（SIEMENS 64排CT，层厚1 mm，间距0.8 mm），导出DICOM格式的数据，将数据导入带有inReach软件的专用计算机，对肺部及支气管影像进行三维重建和导航规划路径。②注册：是指在影像学重建虚拟支气管树中设定6个定位点，在实际导航过程中将根据这些定位点把纤维支气管镜的图像与虚拟支气管树进行匹配。③在CT图像上标定病灶位置和大小，计算机根据病灶位置进行自动路径规划，操作者可对自动规划的路径进行人工校正和修改。④患者仰卧位全麻，喉罩或气管插管通气，胸部置于电磁定位板上方的区域，在患者前胸在胸骨柄、两侧腋中线第8肋间，贴上3个磁极，呈等腰三角形布局。⑤导航操作时先用纤维支气管镜行双侧支气管常规检查，然后将定位导向管连接连接扩展工作通道中插入支气管镜钳道，让导管前段感应器露出气管镜前端，随气管镜到达各个注册点完成注册，随即纤维支气管镜的图像与虚拟支气管树图像完成匹配。⑥注册完成后，将支气管镜置于病灶所在亚段支气管开口，在实时导航下通过操作手柄控制导向管连同扩展工作通道进入病灶部位。固定扩展工作通道，将导向管退出，使用R-EBUS确认病灶，见到支气管周围异常回声可确认导航成功。⑦退出超声探头，使用穿刺针、活检钳、刷检钳通过扩展工作通道进行组织样本的活检，必要时可使用X光监视，活检完成后再次使用R-EBUS确认病灶位置。⑧检查气管腔内有无活动性出血，必要时冰肾上腺素溶液止血处理，全部操作完成后退出扩展工作通道及纤维支气管镜。

### 1.5 病理学检查

刷检标本制作涂片，风干后置于无水酒精中固定，送病理科行脱落细胞学检查，送检验科行找抗酸杆菌检查；支气管灌洗液送病理科行液基细胞学检查；活检钳夹取组织标本经福尔马林液固定、石蜡包埋切片、苏本精-伊红（HE）染色后再进行病理组织学检查，必要时加行免疫组化检查。

### 1.6 统计学方法

采用SPSS 19.0软件进行统计学分析，计数资料采用率（%）表示，组间比较采用*χ*^2^检验；计量资料采用均数±标准差（Mean±SD）表示，组间比较采用*t*检验，以*P*＜0.05为差异有统计学意义。

## 2 结果

### 2.1 电磁导航时间及成功率

所有30例病灶均完成导航定位，导航成功率为100%（30/30），每处病灶导航时间为（25.90±11.29）min；18例患者的手术时间为（95.61±28.74）min。4例患者的7处病灶在导航到达病灶后经R-EBUS确认病灶，再经C臂机监视下进行活检；其余14例患者23处病灶在R-EBUS确认病灶后直接进行活检。

### 2.2 病灶诊断率

30处周围型病灶，每处病灶分别采用细针穿刺抽吸、细胞刷和活检钳取材各4次。阳性诊断率为90%（27/30），其中诊断肿瘤20例，炎症6例，结核1例；阴性诊断率为10%（3/30），诊断为正常肺泡上皮2例，另有1例病灶紧邻血管未行活检。

### 2.3 并发症

术中出现气胸1例，在术中正压通气条件下气胸进展较快，出现血氧饱和度下降，即刻行胸腔闭式引流术，术后1天漏气停止后拔管；术中发生二氧化碳潴留[呼气末二氧化碳分压（endtidal carbon dioxide partial pressure, PET-CO_2_）＞70 mmHg]2例，暂停操作并退出支气管镜，加强机械通气，待PET-CO_2_＜45 mmHg后完成检查；未出现出血、感染、误吸等并发症。

## 3 讨论

ENB技术起源于以色列，于2005年首次在美国应用于临床^[3]^，十余年的临床应用证实该技术是一种安全、可靠、微创、有效的支气管内介入诊疗技术。目前，ENB技术临床应用范围包括：①应用于肺部周围型疑难病灶（如肿瘤、结核、间质性肺病等）的诊断；②应用于纵隔及气管旁淋巴结肿大（如肿瘤转移淋巴结、淋巴结结核、结节病等）的诊断；③扩展应用于肺周围型病灶的介入治疗（如肿瘤消融治疗、肿瘤冷冻治疗等）；④应用于胸腔镜微创手术中的病灶定位^[4]^。

虽然ENB技术是基于患者支气管树空间结构的实时导航，但是该技术对于病灶和导航感应器的空间定位仍然不可避免的存在误差，而R-EBUS可以实时可视化的进行病灶探测和定位。ENB联合R-EBUS可以完成大部分病例导航操作，缩短导航时间，减少了C臂机X线的暴露，可以提高肺周围型病灶的诊断率^[5-7]^。在本组病例中，所有30处病灶均采用ENB联合R-EBUS进行定位，除1例病灶位于肺血管附近分期活检外，其余病灶均顺利完成导航和活检诊断，4例患者的7处病灶联合使用C臂机监视活检，其余14例患者23处病灶未使用C臂机监视。其中发生气胸1例，未使用C臂机监视，考虑病灶位于肺边缘，活检工具穿透胸膜导致气胸，如在C臂机监视下则可清晰观察活检工具的位置，可能能够避免气胸的发生；因此对于紧邻胸膜的周围型病变，C臂机监视下活检更为安全。

ENB技术具备独特的安全、准确、微创的特点^[8-10]^，并可引导外科手术切除病灶^[11, 12]^。在临床上，对于双肺多发性病灶、经皮肺穿刺无法到达或风险过大的部位、严重的肺大泡及肺气肿无法进行肺穿刺的患者，ENB诊断技术更具优势。但是ENB仍然是一项支气管内的介入诊疗技术，其在肺内的到达范围依赖支气管树的分布，部分支气管分布稀疏的肺外周区域依然是ENB的"盲区"，新一代的"CROSS-COUNTRY"技术^[13]^可穿过支气管壁从而实现真正的全肺到达，将进一步拓展ENB技术的临床应用。

ENB检查术一般在全麻气管插管或者喉罩通气状态下进行，本组病例中14例采用气管插管，4例采用喉罩通气，均顺利完成检查，未发生麻醉相关并发症。喉罩通气操作简单，损伤较小，且对支气管镜在气道内的活动限制更小，更适合进行ENB检查术；但是在检查操作时间较长时可能发生管道移位和漏气，需要麻醉医师的严密观察和及时调整。气管插管固定牢固，不容易移位，建议根据患者气道条件尽量选择直径较粗的导管；因为ENB检查术耗时较长，支气管镜对气管导管的阻塞作用比较明显，气管导管内径较细可能导致术中通气不足和二氧化碳潴留。本组病例中出现2例因PET-CO_2_升高达70 mmHg而中断检查，加强通气等待PET-CO_2_降至45 mmHg后恢复操作并顺利完成检查。术中ENB检查术也可以在气道局麻下进行^[14]^，但患者的咳嗽反射和呼吸运动将不可避免地干扰导航操作、延长导航时间并影响导航的成功率，这一问题在临近膈肌的下肺病灶尤为突出，因此局麻下ENB技术的利弊还有待于进一步的临床评估和研究。

ENB技术的安全性和有效性已得到大量研究的证实^[15, 16]^，联合使用R-EBUS进一步提高了肺部病灶的诊断成功率，ENB技术联合微波消融技术治疗肺部肿瘤也已经进入临床研究阶段；但ENB购置成本和高昂耗材价格一定程度上限制了其临床应用。相信，随着ENB技术的不断普及，其使用成本也将会逐步降低，更多的患者将有机会接受ENB的诊断和治疗；伴随着"CROSS-COUNTRY"等新一代导航技术和活检工具的升级应用，ENB技术的应用范围也会得到进一步拓展，ENB将会成为肺周围型病灶诊断治疗中的关键性技术。
